# Generating Patient-Specific Acoustic Simulations for Transcranial Focused Ultrasound Procedures Based on Optical Tracking Information

**DOI:** 10.1109/ojuffc.2023.3318560

**Published:** 2023-09-25

**Authors:** MICHELLE K. SIGONA, THOMAS J. MANUEL, M. ANTHONY PHIPPS, KIANOUSH BANAIE BOROUJENI, ROBERT LOUIE TREUTING, THILO WOMELSDORF, CHARLES F. CASKEY

**Affiliations:** 1 Department of Biomedical Engineering, Vanderbilt University, Nashville, TN 37212, USA; 2 Vanderbilt University Institute of Imaging Science, Nashville, TN 37232, USA; 3 Department of Radiology and Radiological Sciences, Vanderbilt University Medical Center, Nashville, TN 37212, USA; 4 Department of Psychology, Vanderbilt University, Nashville, TN 37240, USA

**Keywords:** Acoustic simulations, optical tracking, transcranial focused ultrasound, ultrasound neuromodulation

## Abstract

Optical tracking is a real-time transducer positioning method for transcranial focused ultrasound (tFUS) procedures, but the predicted focus from optical tracking typically does not incorporate subject-specific skull information. Acoustic simulations can estimate the pressure field when propagating through the cranium but rely on accurately replicating the positioning of the transducer and skull in a simulated space. Here, we develop and characterize the accuracy of a workflow that creates simulation grids based on optical tracking information in a neuronavigated phantom with and without transmission through an *ex vivo* skull cap. The software pipeline could replicate the geometry of the tFUS procedure within the limits of the optical tracking system (transcranial target registration error (TRE): **3.9 ± 0.7** mm). The simulated focus and the free-field focus predicted by optical tracking had low Euclidean distance errors of **0.5±0.1** and **1.2±0.4** mm for phantom and skull cap, respectively, and some skull-specific effects were captured by the simulation. However, the TRE of simulation informed by optical tracking was **4.6±0.2**, which is as large or greater than the focal spot size used by many tFUS systems. By updating the position of the transducer using the original TRE offset, we reduced the simulated TRE to **1.1 ± 0.4** mm. Our study describes a software pipeline for treatment planning, evaluates its accuracy, and demonstrates an approach using MR-acoustic radiation force imaging as a method to improve dosimetry. Overall, our software pipeline helps estimate acoustic exposure, and our study highlights the need for image feedback to increase the accuracy of tFUS dosimetry.

## I. INTRODUCTION

TRANSCRANIAL focused ultrasound (tFUS) is a therapeutic modality successfully demonstrated for drug delivery via blood-brain barrier opening, studying the brain through neuromodulation, and liquefying clots through histotripsy [1]. tFUS is suitable for targeting cortical and deep regions in the brain while maintaining a small, ellipsoidal-shaped volume of concentrated energy on the millimeter scale. The focal size of tFUS transducers requires precise positioning of the transducer relative to the subject’s head, and accurate dosimetry is key to therapeutic outcomes. tFUS procedures have been performed in the magnetic resonance (MR) environment, where MR imaging can be used to assess the transducer’s position and localize the focus through direct measurements of the interaction of ultrasound and tissue such as MR thermometry and MR-acoustic radiation force imaging (MR-ARFI) [2]. A straightforward method to position a spherically curved transducer under MR-guidance is to collect an anatomical scan of the subject so that the transducer surface is visible in the image and the focus location is then estimated using the geometric properties of the transducer [3]. MR-guidance is beneficial for target localization and validation; however, tFUS procedures guided by MR imaging are limited to specific patient populations and facilities with access to MR scanners.

Positioning methods independent of the MR environment are often used to expand patient eligibility for tFUS procedures and reduce associated costs. Transducer positioning methods used outside the MR environment for tFUS procedures include patient-specific stereotactic frames, ultrasound image guidance, and optical tracking. Patient-specific stereotactic frames [4], [5], [6], [7] allow repeatable positioning of a transducer onto a subject’s head with sub-millimeter accuracy. Subject-specific frames require individualized design effort and may require invasive implants unsuitable for procedures with healthy human subjects. Ultrasound image guidance uses pulse-echo imaging during tFUS procedures to position a transducer relative to the skull, determining the distance from the skull using the receive elements of a transducer [5], [8], [9]. Ultrasound-guided experiments result in sub-millimeter spatial targeting error but require large, multi-element arrays and receive hardware that can be expensive. Optical tracking has been used to position transducers in a number of tFUS studies with animals [10], [11], [12], [13], [14] and humans [15], [16], [17], [18] where the focus location of a transducer is defined by a tracked tool, and the position and orientation of the tool are updated in real-time relative to a camera. Optical tracking is completely noninvasive for the subject but has larger targeting error than stereotactic frames and ultrasound image-guidance, with reported accuracy in the range of 1.9–5.5 mm [11], [19], [20], [21], [22], [23]. Partial contribution for the large targeting error from optical tracking may be due to the heterogeneous skull, known to shift and distort the focus, that is currently not encapsulated by the predicted focus location from optical tracking systems.

Compensating for the skull is a challenge because the transducer focus is difficult to predict after interacting with the heterogeneous skull layers. There can be significant differences in skull thickness and shape between subjects [24], thus a uniform correction for the skull is not optimal when working with a large population and a patient-specific approach is preferable. Acoustic properties such as density, speed of sound, and attenuation can be estimated for a single subject from computed tomography (CT) images of the skull [25], [26], [27], [28], pseudo-CTs generated directly from MR images [29], [30], or pseudo-CTs from trained neural networks [31], [32], [33], [34], [35]. The acoustic properties of the skull can then be input into acoustic solvers to simulate the resulting pressure field, temperature rise, or phase and amplitude compensation for a particular subject. There are a number of acoustic simulation tools available [36], [37], where the appropriate simulation method for an application involves a trade-off between simulation speed and accuracy. For transducer positioning methods outside the MR scanner, acoustic simulations have been included in studies to estimate in situ pressure, spatial extent, and heating [38]. Additionally, tFUS procedures guided by MR imaging could benefit from simulations to include subject-specific skull effects and compare the simulated focus with available MR localization tools. Although acoustic simulations are commonly integrated into the preplanning workflow or retrospective analysis of tFUS procedures [39], methods to position the transducer and subject in simulation space are not explicitly defined.

Here, we describe a software pipeline to generate patient-specific acoustic simulation grids informed by geometric transformations that are available during tFUS procedures guided by optical tracking. Our pipeline uses open-source tools that allow the method to be readily implemented. The method creates a transformation from the transducer to the simulated space with three MR scans that can then be repeated outside of the magnet. We demonstrate the software pipeline in neuronavigated experiments with standard tissue-mimicking phantom with and without *ex vivo* skull cap and compare the spatial locations of the simulated focus relative to a ground truth focus detected by MR-ARFI. We evaluate a method that updates the transducer location based on MR-ARFI so that the focus location more closely matches the MR ground truth spatially. By using transforms obtained from optical tracking, the software pipeline streamlines simulation and provides a patient-specific estimate of the in situ pressure. Validation studies with MR-ARFI revealed that although the simulated focus tracks closely with the one predicted by optical tracking, the error in actual focusing is defined by the accuracy limits of optical tracking, which can be compensated but require imaging feedback.

## II. METHODS

### A. PHANTOM CREATION

**FIGURE 1.**
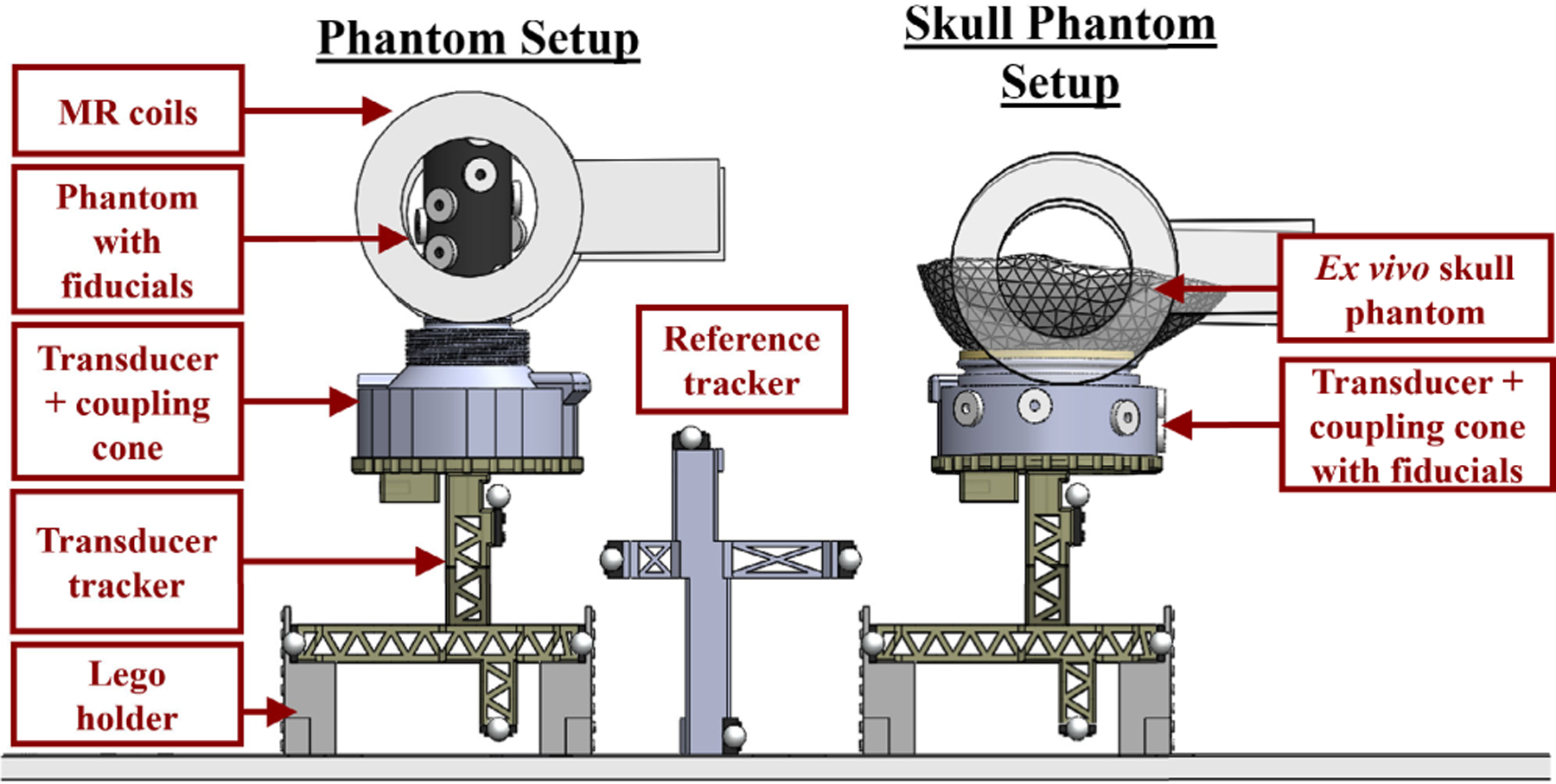
Setup used for phantom and *ex vivo* skull cap phantom experiments. A similar setup was used for both experiments but a different coupling cone with a larger opening was required for the skull phantom setup to ensure sufficient coupling. Fiducials were placed on the coupling cone rather than directly on the *ex vivo* skull, as the fiducials did not adhere well to the rehydrated skull. A lego holder was assembled and used in both setups to elevate the transducer tracker and balance the full assembly.

Agar-graphite phantoms were used for all phantom and *ex vivo* skull cap phantom experiments. Two custom 3D-printed transducer coupling cones designed in-house were used for each setup that better conformed with either the phantom mold or the skull cap, shown in Figure 1. For the phantom setup, the cone was filled with an agar-only layer that consisted of cold water mixed with 1% weight by volume (w/v) food-grade agar powder (NOW Foods, Bloomingdale, IL, USA). The mixture was heated to a boil and once cooled, filled approximately 3/4 of the cone. To create the agar-graphite phantom, a beaker was filled with water mixed with 1% w/v agar powder and 4% w/v 400 grit graphite powder (Panadyne Inc, Montgomeryville, PA, USA). The beaker was heated in the microwave until the contents boiled, and the mixture was removed from heat and periodically stirred to prevent the graphite settling out of solution before the phantom set. Once cooled, the phantom mixture topped off the cone and filled the cylindrical acrylic phantom mold that was adhered to the opening of the transducer coupling cone.

For the *ex vivo* human skull setup, the transducer cone was filled with enough water so that the latex membrane created a flat surface that the skull cap rested on and was secured in place with Velcro straps. The skull cap was rehydrated and degassed in water 24 hours prior to the experiment. The inside of the skull cap was first coated with an agar-only layer detailed above to fill gaps around the sutures of the skull cap. The agar-graphite layer was created using the same methods for the cylindrical phantom and poured to fill the remainder of the skull cap.

### B. NEURONAVIGATION

The optical tracking setup consisted of a Polaris Vicra camera (Northern Digital Inc. (NDI), Waterloo, Ontario, CAN), custom transducer and reference trackers, and an NDI stylus tool. The custom trackers were designed with four retroreflective spheres, as recommended by NDI’s Polaris Tool Guide, and printed in-house. The geometry of each tool was defined using NDI 6D Architect software. A tracked tool’s position and orientation was updated in real-time via the Plus toolkit [40], which streamed data from the optical tracking camera to 3D Slicer [41] and interfaced with the OpenIGTLink [42] module. The streamed data updated the transducer’s focus position and projected a point onto an image volume through a series of transformations to traverse coordinate systems associated with the image (I), physical (P), tracker (T), and ultrasound (U) spaces. Transformations between two coordinate systems are represented as ^*B*^*T*_*A*_, or a transform from coordinate system A to coordinate system B.

The creation and calibration of required transformations have been established by previous work from our group and others [19], [20], [43]. Briefly, six doughnut-shaped fiducials (MM3002, IZI Medical Products, Owings Mills, Maryland, USA) with an outer diameter of 15 mm and an inner diameter of 4.5 mm were placed around the phantom mold or the transducer cone. The calibrated NDI stylus tool was placed in each fiducial to localize the points in physical space. The corresponding fiducials were collected in image space from a *T*_1_-weighted scan (FOV: 150 mm × 170 mm × 150 mm, voxel size: 0.39 mm × 0.50 mm × 0.39 mm, TE: 4.6 ms, TR: 9.9 ms, flip angle: 8°) acquired on a 3T human research MRI scanner (Ingenia Elition X, Philips Healthcare, Best, NLD) with a pair of loop receive coils (dStream Flex-S; Philips Healthcare, Best, NLD). The fiducials were registered with automatic point matching through SlicerIGT’s [44] Fiducial Registration Wizard and resulted in the physical-to-image space transformation (^*I*^*T*_*P*_). The root mean square error distance between the registered fiducials (i.e the Fiducial Registration Error) was recorded from the module [45].

Two custom trackers were used: a reference tracker as a global reference that allowed the camera to be repositioned as needed during experiments, and a transducer tracker. The transducer tracker’s position and orientation in physical space was defined relative to the reference tracker and reported by the NDI camera as a transformation matrix, ^*P*^*T*_*T*_. A single-element, spherically curved transducer (radius of curvature = 63.2 mm, active diameter = 64 mm, H115MR, Sonic Concepts, Bothell, Washington, USA) was used for all experiments, operated at the third harmonic frequency of 802 kHz. The geometric focus of the transducer was calibrated relative to the transducer’s tracker by attaching a rod with an angled tip, machined so that the tip was at the center of the focus [19]. The rod was pivoted about a single point to create the transformation ^*T*^ *T*_*U*_, which is a translation of the transducer’s focus location from the transducer’s tracker. The focus location was visualized using a sphere model created with the ‘Create Models’ module in Slicer, where the radius was set by the expected full-width at half-maximum focal size of the transducer at 802 kHz. ^*T*^ *T*_*U*_ of this transducer was validated in previous work [13] with MR thermometry, that included a bias correction of [X,Y,Z] = [2,0,4] mm where the z-direction is along the transducer’s axis of propagation.

### C. SIMULATION PIPELINE

**FIGURE 2.**
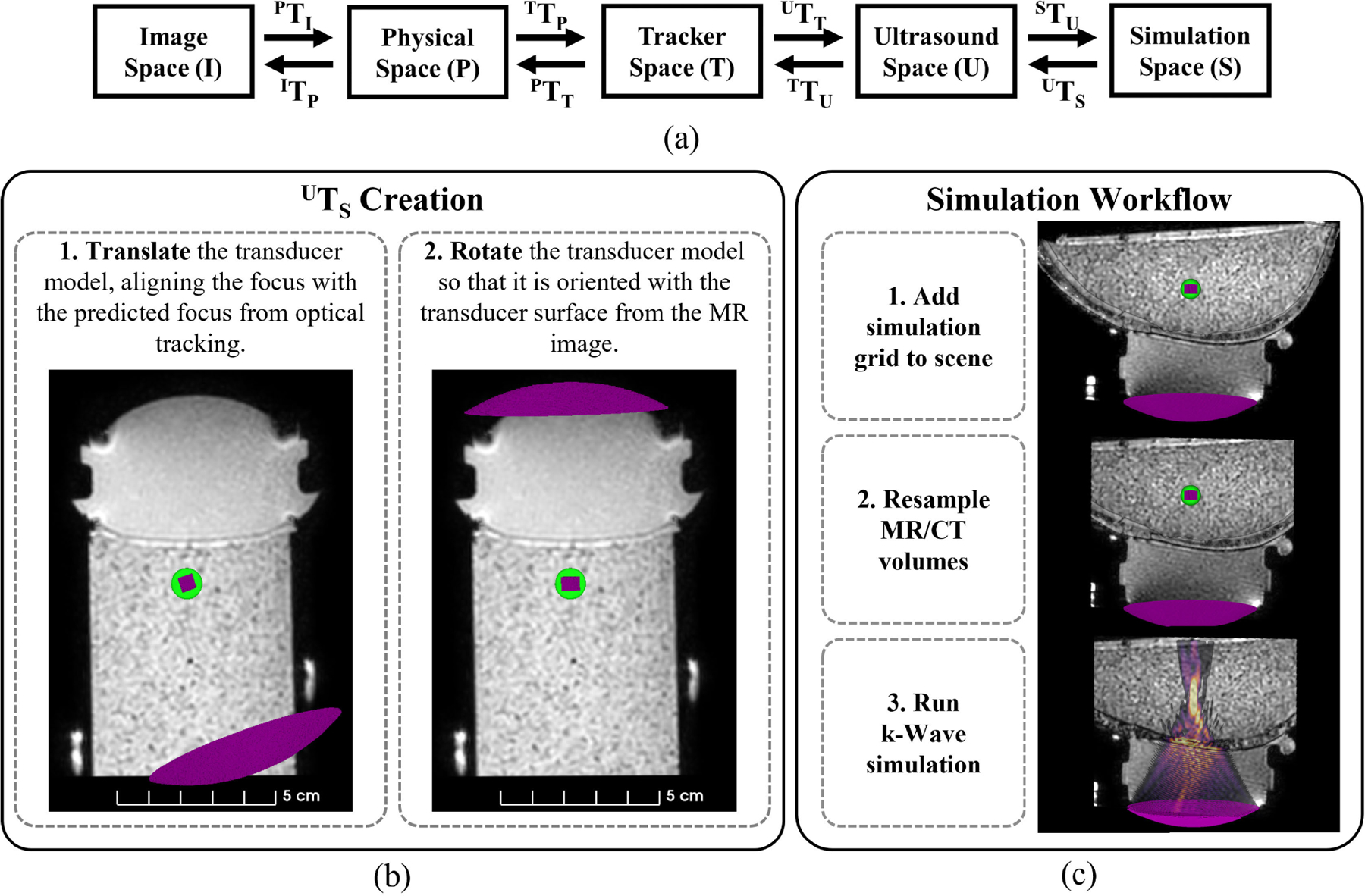
The full transformation hierarchy for optical tracking informed simulations are shown with steps detailing the workflow. All coordinate spaces and their corresponding transformations to navigate between each space are listed (a). Two steps are required to create the transformation between ultrasound and simulation space (b). The green sphere model represents the predicted focus from optical tracking and the pink cube denotes the geometric focus of the transducer model. Once ^*U*^*T*_*S*_ is created, the simulation workflow consists of applying the transformation hierarchy to the transducer model, resampling the images, and exporting the files to MATLAB to run the simulation (c). Transformation ^*U*^*T*_*S*_ can be applied to any neuronavigation scene with the same transducer, transducer tracker, and transducer model.

A new transformation, ^*U*^*T*_*S*_, was required to add on to the transformation hierarchy and transform the simulation grid (S) to the ultrasound coordinate system shown in Fig 2a. First, a model of the transducer was created using the k-Wave function makeBowl, with a cube centered at the geometric focus location that assisted with visualization. The model was imported into Slicer as a NIFTI file and was manually translated so that the center of the model’s cube was aligned with the predicted focus from optical tracking shown in step 1 of Fig. 2b. Because the transducer surface was visible in the *T*_1_-weighted image due to the large signal from the water-filled cone, the transducer model was manually rotated until the model matched the orientation of the transducer surface from the MR image like in step 2 of Fig. 2b. The transducer model may be slightly offset from the transducer surface in the *T*_1_-weighted image, especially if a bias correction was applied in the previous transformation ^*T*^ *T*_*U*_, as was the case in Fig 2b. This calibration was performed with three separate *T*_1_-weighted images and the resultant transformations, a separate transformation for each rotation and translation, was averaged to create the final transformation, ^*U*^*T*_*S*_. The creation of ^*U*^*T*_*S*_ only needs to be performed once and can be added to any scene coupled with the same transducer model, transducer, and transducer tracker.

The workflow to incorporate simulations using optical tracking data is detailed in Fig. 2c. ^*U*^*T*_*S*_ was added to the saved Slicer scene that contained the neuronavigation data from eight phantom experiments and three *ex vivo* skull cap phantom experiments. First, the transformation hierarchy from the saved scene was used to transform the transducer model into image space. Next, MR/CT volumes were resampled with the ‘Resample Image (BRAINS)’ Module so that the voxel and volume sizes matched the simulation grid. For skull phantom experiments, a CT image was acquired on a PET/CT scanner (Philips Vereos, Philips Healthcare, Best, NLD) with an X-ray voltage of 120 kVp and exposure of 300 mAs (pixel resolution: 0.30 mm and slice thickness: 0.67 mm) and was reconstructed with a bone filter (filter type ‘D’). The CT image was manually aligned to match the orientation of the *T*_1_-weighted MR volume (parameters described in II-B) and then rigidly registered using the ‘General Registration (BRAINS)’ module in Slicer. The simulation grid containing the transducer model and resampled MR/CT volumes were saved as NIFTI file formats to use for simulations.

All simulations were performed using the MATLAB acoustic toolbox, k-Wave [46], with a simulation grid size of [288,288,384] and isotropic voxel size of 0.25 mm, where we maintained greater than 7 points per wavelength in water for simulation stability [47]. For phantom setups, the simulation grid was assigned acoustic properties of water selected from the literature [48] and shown in Table 1 with the exception of absorption. We previously measured the attenuation in agar-graphite phantoms as 0.6 dB/cm/MHz [20] and assumed absorption was a third of the attenuation value [48], [49]. Thus, all pixels in the agar-graphite layer of the phantom were assigned 0.2 dB/cm/MHz for *α*_*tissue*_. For simulations with the skull cap, the skull was extracted from the CT image using Otsu’s method [50], [51] and a linear mapping between Hounsfield units and bone porosity [25] was used to derive acoustic properties of the skull. A ‘‘tissue’’ mask was created from the agar-graphite phantom using Slicer’s ‘Segment Editor’ module and assigned as *α*_*tissue*_. For all simulations, the RMS pressure was recorded and the focus location was defined as the maximum pixel. For cases where the maximum pressure was at the skull, as previously observed in simulations with this transducer [52], the tissue mask was eroded using the ‘imerode’ function in MATLAB to exclude pixels closest to the skull. The eroded mask was applied to the simulated pressure field to select the maximum in situ pressure.

### D. MR VALIDATION

The focus was localized using MR-acoustic radiation force imaging (MR-ARFI) described in prior work [52], measuring displacement induced by the transducer in both phantom and *ex vivo* skull cap setups. MR-ARFI was acquired using a 3D spin echo sequence with trapezoidal motion encoding gradients (MEG) of 8 ms duration and 40 mT/m gradient amplitude strength (voxel size: 2 mm × 2 mm × 4 mm, TE: 35 ms, TR: 500 ms, reconstructed to 1.04 mm × 1.04 mm × 4 mm). Only positive MEG polarity was required to measure sufficient displacement in phantoms. With the skull, two scans were acquired with opposite MEG polarity. A low duty-cycle ultrasound sonication was used for all ARFI scans (8.5 ms per 1000 ms/2 TRs, free-field pressure of 1.9 MPa for phantoms and 3.2 MPa for *ex vivo* skull cap).

### E. ERROR METRICS

Metrics were chosen that assessed the targeting accuracy of the neuronavigation system and the spatial accuracy of the simulated pressure fields. First, the target registration error (TRE) of our neuronavigation system was determined from the Euclidean distance between the center of the predicted focus from optical tracking and the center of the MR-ARFI focus for all experiments, or *TRE*_*Opti,ARFI*_. It was assumed that the predicted focus from optical tracking was positioned at the desired target. The MR measured focus from the displacement image was manually selected by adjusting the visualization window and level tools of the volume in 3D Slicer to pick the approximate maximum pixel within the center of the focus. To compute the accuracy of the focus simulated based on optical tracking geometry, the distance between the center of the simulated focus and the optically tracked focus was calculated (*Error*_*Opti,Sim*_). The center of the simulated focus was chosen from the centroid of the volume using 3D Slicer’s ‘Segment Statistics’ module, thresholded at half of the maximum pressure to create a segmentation of the focus. Finally, we evaluated the simulated pressure field compared to our ground truth MR measurement (*Error*_*Sim,ARFI*_ ). For all error metrics, separate axial and lateral components were evaluated. The axial component was determined from the offset between the two foci along the transducer’s axis of propagation and the lateral component was the Euclidean norm of the X and Y directions. The focus locations were selected in 3D Slicer where volumes are automatically upsampled for visualization, thus the metrics are reported at higher precision than the simulation grid.

To achieve better agreement between experimental and simulated estimates of the focus, the distance vector between the predicted focus from optical tracking and the MR-ARFI focus was applied as a translation to the transducer model and re-simulated. The spatial error was quantified between the displacement image and the updated simulation results (*Error*_*SimUpdated,ARFI*_ ).

## III. RESULTS

### A. PHANTOM RESULTS

**FIGURE 3.**
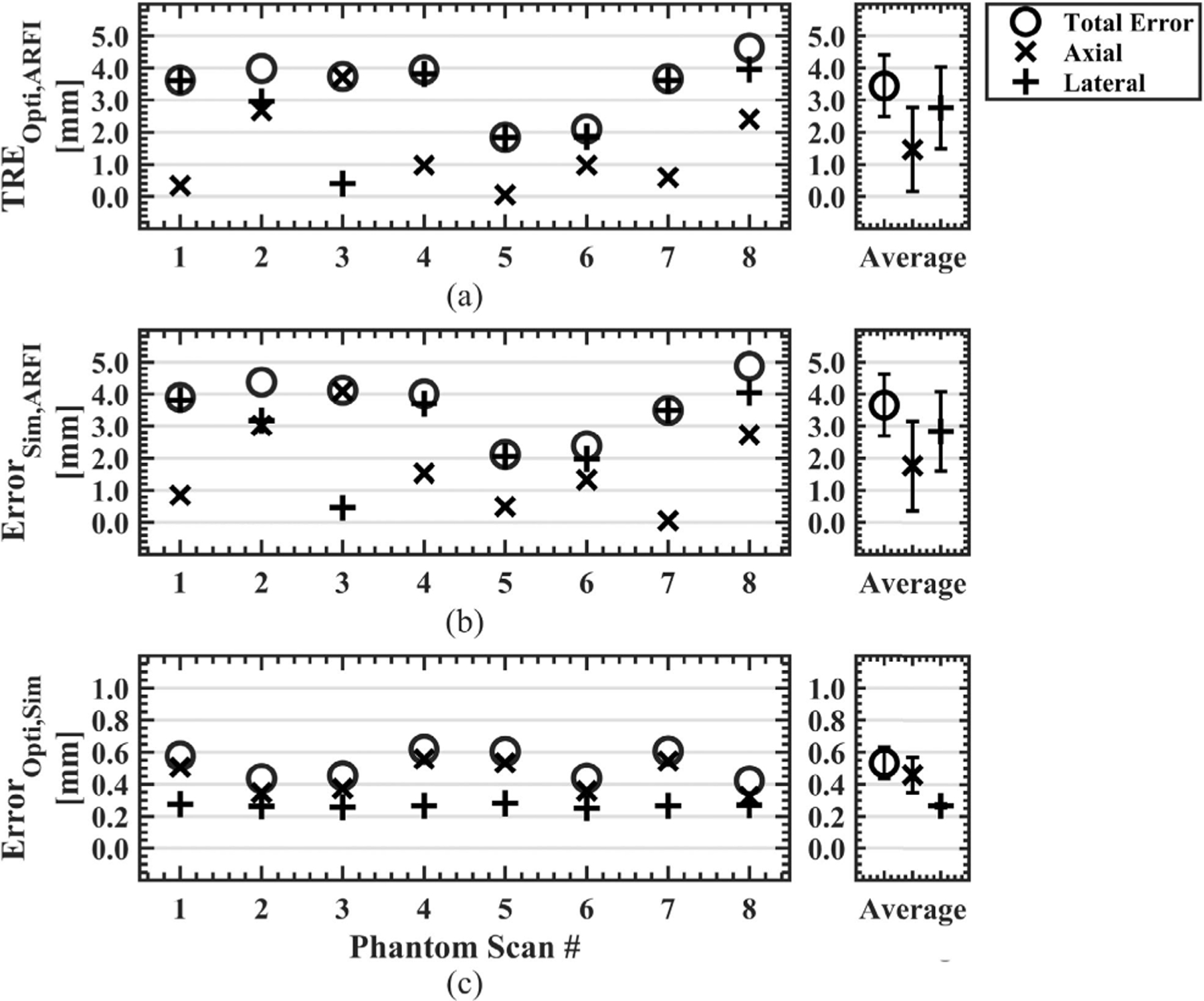
Error metrics to quantify the error of the optical tracking system and simulation workflow are shown. Axial and lateral components were separately evaluated for all error metrics, and the average of all phantom scans is shown on the right. The Euclidean distance between the predicted focus from optical tracking and the manually selected center of the MR-ARFI focus was calculated to obtain the targeting accuracy of the optical tracking system (a). The centroid of the simulated pressure field, thresholded at 50% of the maximum pressure, was compared against the MR-ARFI focus (b). The optically tracked and simulated foci defined the pipeline accuracy, where the total error was less than 1 mm for all scans and largely observed in the axial direction (c). (TRE = target registration error, Opti = optically tracked focus, ARFI = acoustic radiation force imaging, Sim = simulated focus.)

**FIGURE 4.**
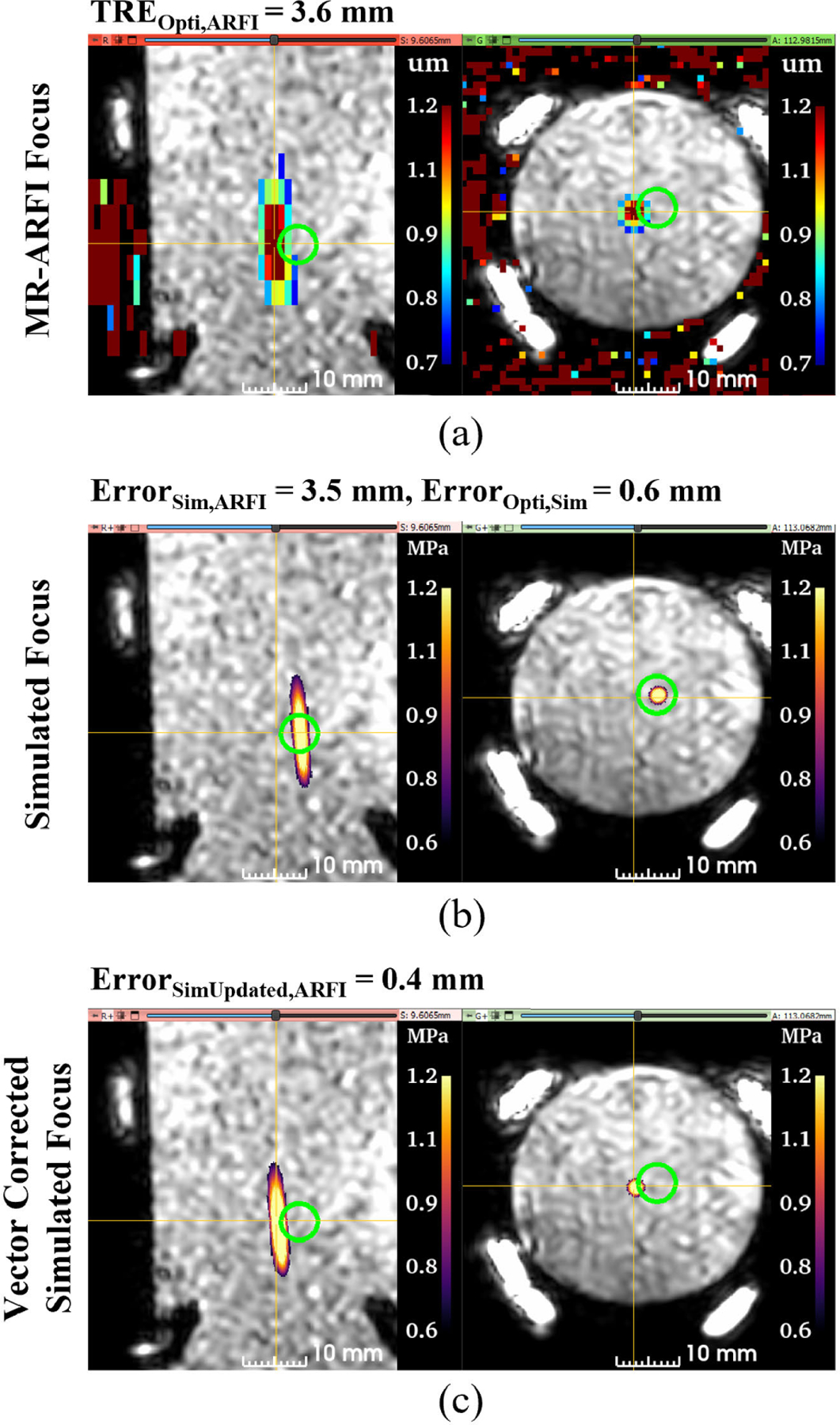
Representative case to demonstrate the spatial differences between the foci from each volume. All slices are centered at the MR-ARFI focus for comparison. The MR-ARFI focus is offset by 3.6 mm from the predicted focus from optical tracking, represented by the green sphere model (a). The simulated focus, thresholded at 50% of the maximum pressure, is slightly offset axially compared to the optically tracked focus location (b). With vector correction, the simulated focus now spatially aligns with the MR-ARFI focus (c). (TRE = target registration error, Opti = optically tracked focus, ARFI = acoustic radiation force imaging, Sim = simulated focus.)

For all phantom data sets (N=8), the target registration error of the optical tracking system, or *TRE*_*Opti,ARFI*_, was 3.4 ± 1.0 mm. *TRE*_*Opti,ARFI*_ and the separated axial and lateral error components are plotted in Figure 3a for each data set and the average for all data sets. The mean axial and lateral *TRE*_*Opti,ARFI*_ was 1.5 ± 1.3 mm and 2.8 ± 1.3 mm, respectively. Similarly, *Error*_*Sim,ARFI*_ is plotted in Figure 3b and describes the error between the simulated pressured field positioned from optical tracking data compared to the MR-ARFI focus location. Average *Error*_*Sim,ARFI*_ was 3.7 ± 1.0 mm with 2.8±1.2 mm of lateral error and 1.8±1.4 mm of axial error. Figure 3c compared the error between the predicted and simulated foci, *Error*_*Opti,Sim*_, with a group average of 0.5±0.1 mm, with errors of 0.4±0.1 mm and 0.3±0.0 mm in the axial and lateral directions. Updated simulations from the distance vector correction were less than a millimeter, reducing the original simulation error of *Error*_*Sim,ARFI*_ from 3.7 ± 1.0 to 0.5 ± 0.1 mm for *Error*_*SimUpdated,ARFI*_. Slices from phantom dataset #1 centered about the MR-ARFI focus are shown in Figure 4 to demonstrate the spatial differences between each volume where Figure 4a shows the targeting error, Figure 4b shows the simulation pipeline error, and Figure 4c shows the updated simulation results which better spatially match the MR-ARFI focus in Figure 4a.

### B. EX VIVO SKULL PHANTOM RESULTS

**FIGURE 5.**
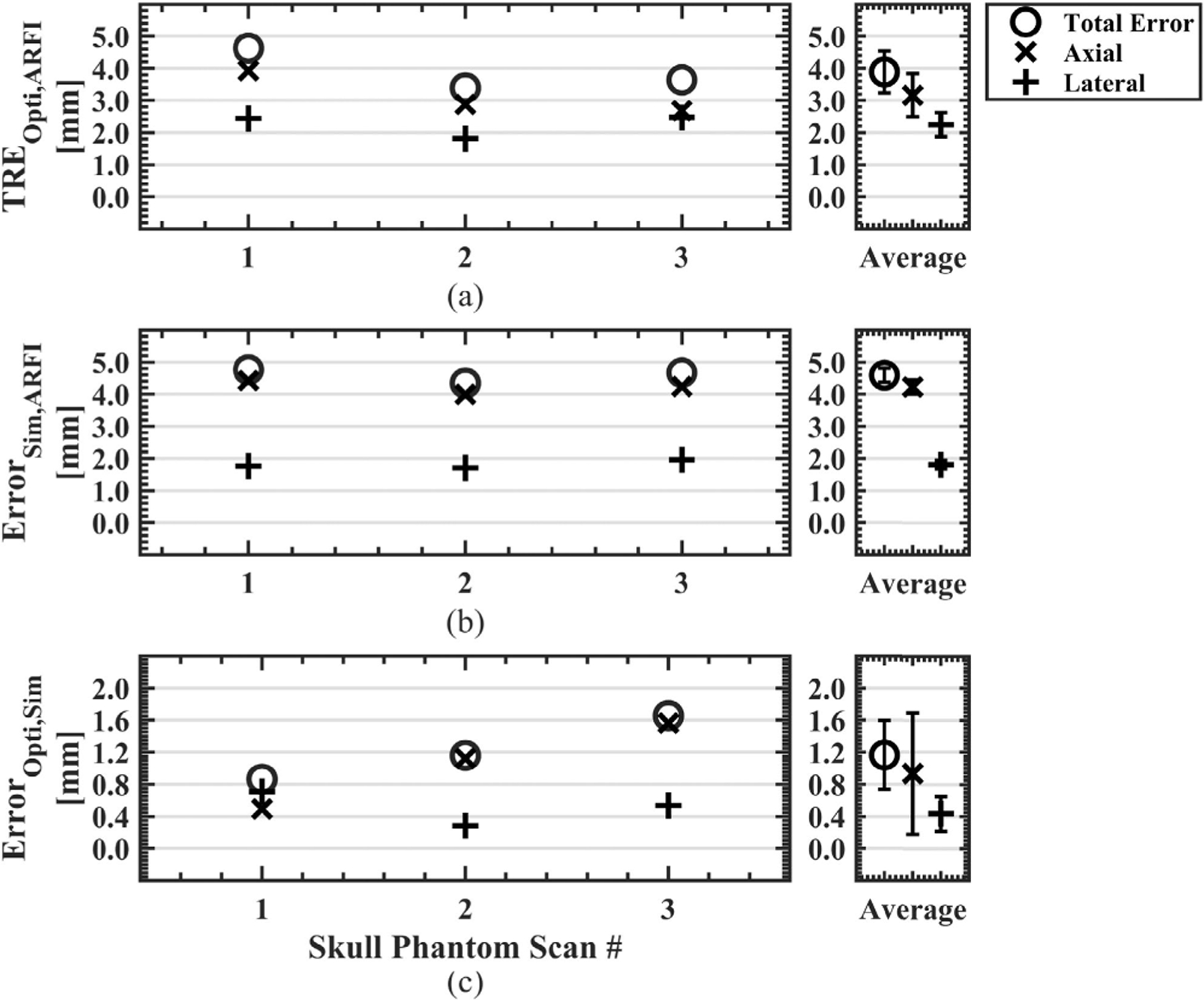
Error metrics shown to quantify the accuracy of the simulation workflow for transmission through an *ex vivo* skull cap. A larger axial component error was noted for the optical tracking system error compared to phantom data sets without the skull (a). Similarly, the simulated focus compared to the MR-ARFI focus was largely comprised of axial error (b). The error between the predicted and simulated foci increased from 0.5 ± 0.1 mm in phantoms to 1.2 ± 0.4 mm when incorporating the skull. (TRE = target registration error, Opti = optically tracked focus, ARFI = acoustic radiation force imaging, Sim = simulated focus.)

The simulation pipeline was further evaluated with transmission through an *ex vivo* skull phantom at three transducer orientations. Mean *TRE*_*Opti,ARFI*_ was 3.9 ± 0.7 mm with 2.2 ± 0.4 mm and 3.2 ± 0.7 mm of lateral and axial error, respectively as shown in 5a. Figure 5b shows *Error*_*Sim,ARFI*_ had larger axial error and total error than *TRE*_*Opti,ARFI*_. Average *Error*_*Sim,ARFI*_ was 4.6 ± 0.2 mm comprising of 4.2 ± 0.2 mm error axially and 1.8 ± 0.1 mm error laterally. The averaged *Error*_*Opti,Sim*_ in 5c increased from 0.5 ± 0.1 mm in phantoms to 1.2±0.4 mm when incorporating the skull, with individual axial and lateral components of 1.1±0.5 mm and 0.5±0.2 mm for the skull phantom, respectively. The distance vector correction improved the simulated focus location from an *Error*_*Sim,ARFI*_ of 4.6 ± 0.2 mm to *Error*_*SimUpdated,ARFI*_ of 1.2 ± 0.4 mm. Similarly, axial and lateral errors of *Error*_*Sim,ARFI*_ improved from 4.2±0.2 mm and 1.8±0.1 mm to *Error*_*SimUpdated,ARFI*_ errors of 0.9 ± 0.8 mm and 0.4 ± 0.2 mm, respectively.

**FIGURE 6.**
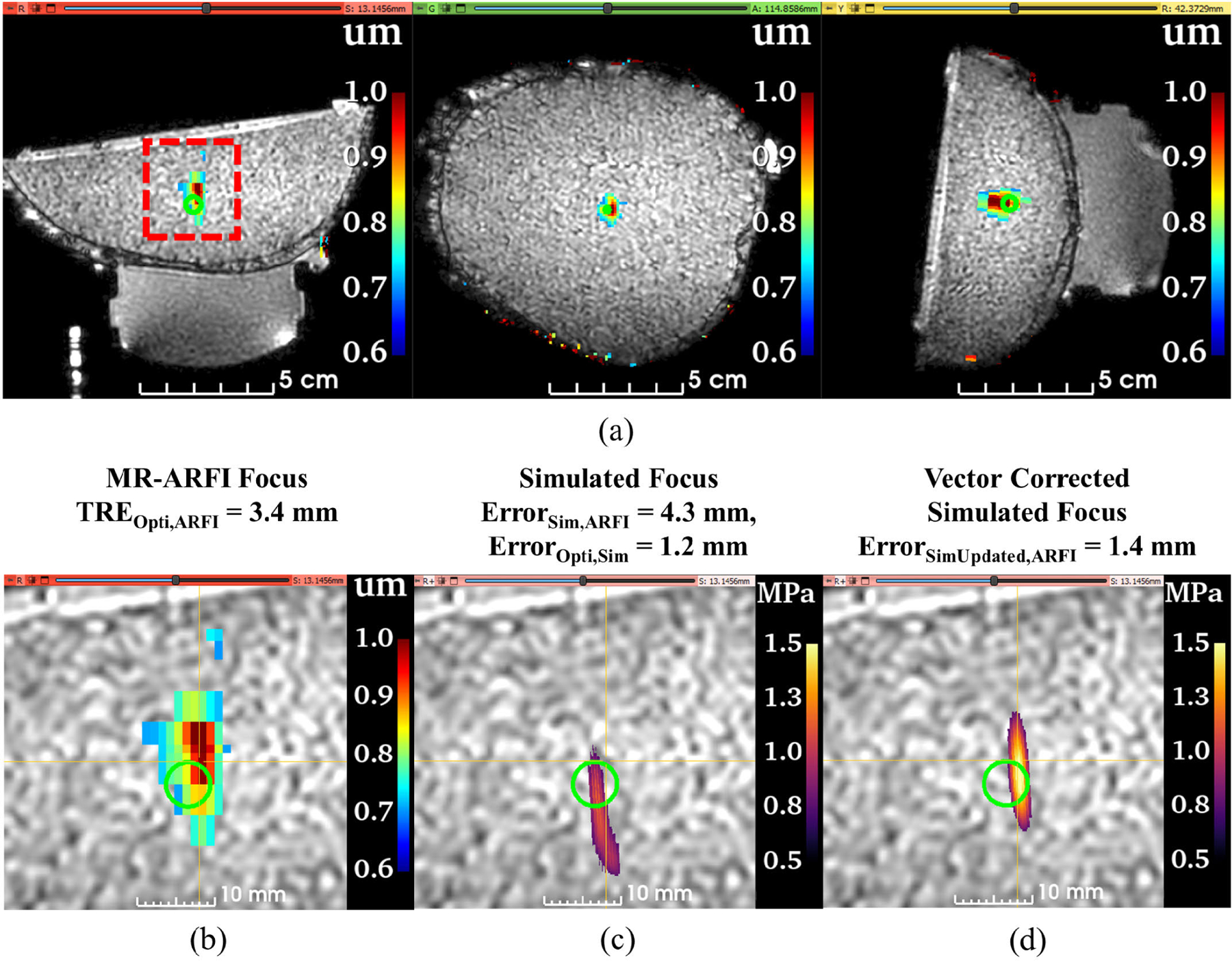
A representative case from *ex vivo* skull phantom data set #2 to demonstrate the accuracy of the simulation workflow when transmitting through human skull. The full field of view of the T1-weighted image is shown with the MR-ARFI displacement map overlaid (a). Slice views in b-d are zoomed about the red dashed box in the red slice for easier visualization. Enlarged view of the MR-ARFI displacement map and the predicted focus from optical tracking (green sphere model) (b). Because the slice views are centered on the MR-ARFI focus, the center of the simulated focus is not visible, emphasizing the offset between the MR measurement and simulated focus (c). However, the spatial offset is largely corrected after re-simulating with a vector correction applied (d). (TRE = target registration error, Opti = optically tracked focus, ARFI = acoustic radiation force imaging, Sim = simulated focus.)

Figure 6a shows MR-ARFI displacement maps through an *ex vivo* skull cap from skull phantom data set #2. Enlarged views showed the volume used to calculate *TRE*_*Opti,ARFI*_ in Figure 6b. An axial shift is noted between the predicted focus and simulated focus in Figure 6c and because slices are centered at the MR-ARFI focus location for comparison, we do not observe the center of the simulated focus location as *Error*_*Sim,ARFI*_ is 4.3 mm for this example case. However, the updated simulation results from vector correction reduced the error to 1.4 mm in 6d.

**FIGURE 7.**
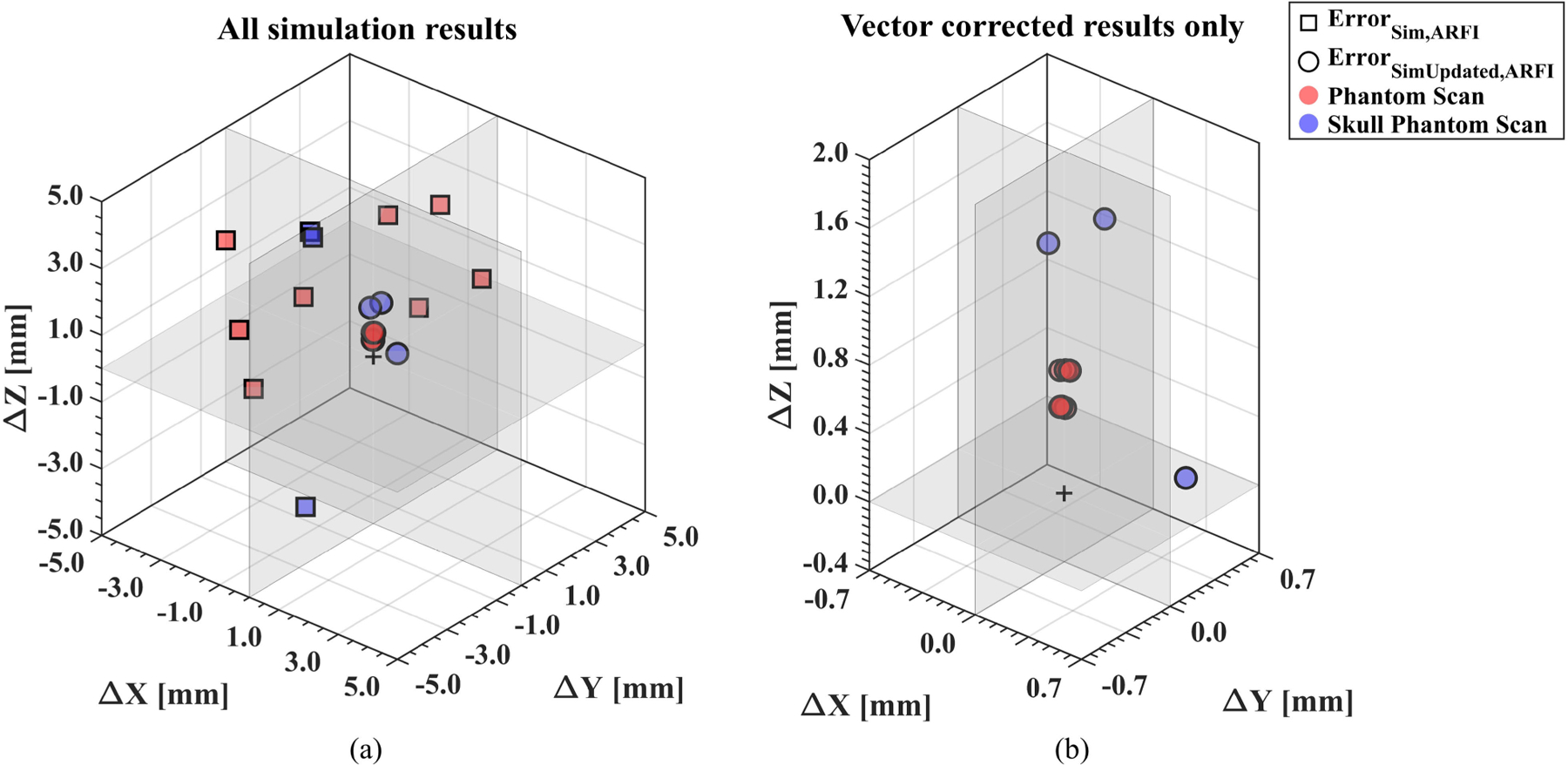
Initial simulation results compared to the vector-corrected simulation results demonstrate that error could be improved with imaging feedback. For each data set, the vector components between the simulated focus and the MR-ARFI focus are shown. The initial simulation results (square markers) show large errors in both the lateral (X and Y) and axial (Z) directions observed in both phantom (red) and skull phantom (blue) data sets (a). The same plot is enlarged to observe the vector-corrected simulation results (circle markers). The overall error was reduced, with the remaining error largely in the axial direction (b). (ARFI = acoustic radiation force imaging, Sim = simulated focus.)

The ground truth focus location from the MR-ARFI displacement map ([X,Y,Z] = [0,0,0]) is plotted against the simulated focus location before and after vector correction, where the improvement can be visualized in Figure 7 for both phantom and skull phantom data sets. The initially simulated foci show the error is not biased in a given direction compared to the MR-ARFI focus pltoted in Figure 7a. The simulation results demonstrate there is not a uniform correction that would improve *TRE*_*Opti,ARFI*_ or *Error*_*Sim,ARFI*_. For the vector-corrected cases in Figure 7b, the remaining error further demonstrates the offset attributed to either absorption in phantoms or the medium properties due to the skull.

## IV. DISCUSSION

Neuronavigation using optical tracking provides a noninvasive approach to position a transducer about a subject’s head during tFUS procedures independent of the MR scanner for guidance. For transcranial applications, the aberrating skull displaces the focus from the intended target but because of subject skull variability, this offset is difficult to predict in real-time. Acoustic simulations can predict attributes of the focus after interacting with the skull, but methods to position the transducer in the simulated space are not explicitly defined or achieved using ad hoc methods. Here, we proposed a method that uses transformations from optical tracking to position a transducer model in simulations representative of the transducer position during tFUS procedures. Metrics were chosen to quantify the spatial error of the simulated focus compared with the optically tracked or MR-measured focus. A correction method to update the transducer model was proposed to address inherent errors of using optical tracking to set up the pipeline. This simulation pipeline can be a tool to accompany optically tracked tFUS procedures outside of the MR environment and provide subject-specific, in situ estimates of the simulated pressure fields.

We first assessed the accuracy of our simulation workflow informed by optical tracking data for neuronavigated FUS experiments. A low error between the predicted focus from optical tracking and the simulated focus (*Error*_*Opti,Sim*_) was observed in both phantom (0.5 ± 0.1 mm) and skull phantom data sets (1.2 ± 0.4 mm). The larger *Error*_*Opti,Sim*_ with the skull comprised of 1.1 ± 0.5 mm of axial error which can be attributed to skull-specific effects captured in simulation. Although we did notice minor focal shifts with the presence of the *ex vivo* skull, other simulation studies of *ex vivo* and in situ scenarios indicate larger focal shifts may be expected depending on the skull characteristics, brain target, and transducer properties [11], [22], [53]. This study largely focused on the spatial error of the simulation workflow where we used previously validated acoustic parameters [48]. However, when considering this workflow for dosimetry estimates, importance should be placed on parameter selection, as a sensitivity analysis from Robertson et al. demonstrated the importance of accurately accounting for the speed of sound [54]. Coupled with Webb et al., noting the relationship of speed of sound and HU may vary between X-ray energy and reconstruction and velocity of the human skull [55].

Although low *Error*_*Opti,Sim*_ is promising for reproducing neuronavigated tFUS setups *in silico*, our accuracy assessment comparing the simulated pressure field to our MR measurement (*Error*_*Sim,ARFI*_ ) demonstrates that the simulation grids are only as accurate as the error of the optical tracking system (*TRE*_*Opti,ARFI*_ ). In our specific case, the lowest target registration error used to define our optical tracking system error was 3.5 ± 0.7 while *Error*_*Sim,ARFI*_ was either similar (3.7±1.0 mm in phantoms) or worse (4.6±0.2 mm in skull phantoms) than *TRE*_*Opti,ARFI*_. Improving the targeting accuracy of a system could include measures such as increasing the number of fiducials, optimizing fiducial placement, and reducing fiducial localization error [56], [57].

When translating the neuronavigation setup and simulation workflow used in this study for human subjects, there should be careful consideration regarding fiducial placement and transducer positioning. Fiducial placement should surround the target so that the centroid of the fiducial markers is as close to the target as possible [56], similar to the fiducial placement during the phantom scan experiments. However, spatially distributing fiducials around targets in the brain is not always feasible in practice. Previous neuronavigation experiments with human subjects identified skin blemishes or vein structures that can be reproducibly selected for multiple experiments [58] or placed near prominent features such as behind the ears and above the eyebrows [59]. Nonhuman primate experiments without MR guidance have created custom mounts to repeatably place fiducials on the subject’s head [13]. For nonhuman primates, Boroujeni et al. attached the fiducials to the headpost which may translate to a helmet design similar to the experimental setup of Lee et al. [58] or custom headgear of Kim et al. [59].

Because fiducial placement is limited for human subjects, we may expect a larger targeting error when translating the neuronavigation workflow for human studies. Additionally, the error may depend on the target in the brain. For example, it may be expected that deeper regions in the brain that are further from the centroid of the fiducial markers result in larger targeting errors [56]. While we do not know the exact angle of approach and target depths from Xu et al. *in silico* study, it was observed that targets located in the frontal and temporal lobes had larger axial offsets compared to the occipital and parietal lobes, observing cortical and deeper regions had larger errors due to the skull interfaces [22]. For acoustic simulations, pseudo CT images have been proposed as alternatives to acquiring CT images that expose human subjects to ionizing radiation [29], [30], [31], [32], [33], [34], [35], [60], [61]. We do not anticipate any technical challenges adapting pseudo CT images into the simulation workflow proposed in this study.

Existing optical tracking systems can have errors greater than the expected focal size of the transducer, which can result in targeting undesired regions of the brain. Thus, we developed a correction method to reduce the targeting accuracy and update the transducer model in simulation space so that the resulting pressure field spatially matches the MR-ARFI focus. For our work, we informed the vector correction using the center of the MR-ARFI focus, but this correction method can be explored with other imaging methods such as MR thermometry [62]. The updated simulation grids can be used for further analysis of tFUS procedures, as accurate placement of the simulation models will be useful when adapting the workflow for multi-element arrays to perform steered simulations. However, we note that the vector correction applied in this work assumes that the error can be updated solely by a translation, where rotational correction was not explored in the scope of this work.

The simulation workflow presented here is dependent on the initial calibration at the MR scanner to create the transformation required to add the simulation coordinate space to the existing optical tracking transformation hierarchy. This poses a limitation for transducers that are not MR-compatible that require adapting setups for calibration and validation of setups fully independent of the MR scanner. One proposed method could leverage previous methods from Chaplin et al. and Xu et al. to identify the transducer surface using an optically tracked hydrophone and assess the targeting accuracy in a water bath [20], [22]. Chaplin et al. described methods to produce an optically tracked beam map that could be expanded to localize the transducer surface using back projection [63] for simulations. The targeting accuracy of the optical tracking system could then be assessed using recent methods by Xu et al. using a water bath, hydrophone, and CT image, rather than MR localization methods. A fully independent method from the MR scanner becomes increasingly important when considering translation to human subjects where some MR tools such as MR-ARFI still require further development before implementation in a clinical setting.

## V. CONCLUSION

In our study, we described a workflow to integrate acoustic simulations with optically tracked tFUS setups. Simulations from our pipeline were validated with MR measurements and found comparable results with the predicted focus from optical tracking. However, this pipeline is limited by the targeting accuracy of the optical tracking system. To improve estimates, we proposed a vector correction method informed by MR-ARFI to update the transducer model in the simulation grid, which resulted in the improved spatial representation of the ground truth focus. This pipeline can be applied to existing tFUS neuronavigation setups using two open-source tools, aiding in the estimation of in situ characteristics of the ultrasound pressure field when MR-guidance is unavailable.

## Figures and Tables

**TABLE 1.** Acoustic properties used for all simulations.

Speed of Sound (*m*/*s*)	Density (*kg*/*m*^3^)	Absorption (*dB*/*cm*/*MHz*)
*c*_*water*_ = 1500	*ρ*_*water*_ = 1000	*α*_*water*_ = 0 *α*_*tissue*_ = 0.2
*c*_*bone*_ = 3100	*ρ*_*bone*_ = 2200	*α*_*bone, min*_ = 0.02 *α*_*bone, max*_ = 2.7
